# Management of Patella Dislocation in Say-Barber-Biesecker-Young-Simpson's Syndrome: A Report of Two Cases

**DOI:** 10.1155/2018/6415713

**Published:** 2018-04-03

**Authors:** Meni Mundama, Serge Ayong, Renaud Rossillon

**Affiliations:** Department of Orthopaedic Surgery, Clinique Saint-Pierre Ottignies, Avenue Reine Fabiola 9, 1340 Ottignies, Belgium

## Abstract

Say-Barber-Biesecker-Young-Simpson's syndrome is one of the Ohdo-like syndromes. It is a very rare congenital condition that is commonly defined by its main clinical features that are blepharophimosis, ptosis, mental retardation, and delayed motor development. They are often associated with skeletal manifestations that are joint laxity, long thumbs and toes, and hypoplastic and/or dislocated patellae. To our knowledge, the available literature does not report any case where attention is drawn to management of skeletal aspect of this specific syndrome, especially surgically. We report 2 cases of SBBYS syndrome with patellar dislocation that we followed for 11 years. One case (with bilateral dislocation) was managed conservatively, and the other (with unilateral dislocation) underwent conservative and surgical treatment. Both had good functional outcome at follow-up. This experience shows that patellar abnormality in this condition can be efficiently addressed conservatively and/or surgically with satisfying results.

## 1. Introduction

Say-Barber-Biesecker-Young-Simpson (SBBYS) variant of Ohdo's syndrome is a very rare condition known to feature blepharophimosis, ptosis, mental retardation, delayed motor milestones, impaired speech, dental abnormalities, and hearing dysfunction. Cryptorchidism and scrotal hypoplasia are reported in male patients. Some skeletal characteristics are usually associated: joint laxity, abnormally long thumbs and great toes, and dislocated or hypoplastic patellae. At birth, early signs are hypotonia and feeding problems. Other features of variable frequency are described, such as cardiac defects and abnormal thyroid structure or function [1, 2].

This condition is related to KAT6B gene mutation (OMIM# 603736) [2].

To our knowledge, among SBBYS syndrome cases with patellar dislocation, no surgical case has been reported to date.

For more than 11 years, we followed two boys with SBBYS syndrome presenting patellar dislocation. Initially, both were managed conservatively. One patient finally underwent a surgical procedure aiming to optimise his walking and standing function. We have no records of earlier publication on any of the two cases.

## 2. Case Presentation

### 2.1. Case 1

Patient 1 is a boy born in 2002, followed by the senior author since age 3 (Table 1). He then featured bilateral blepharophimosis and ptosis, dental abnormalities, hypogonadism, and heart defects (pathologic valve and interatrial communication). He had psychomotor delay and presented skeletal anomalies: left side metatarsus adductus with reducible hindfoot valgus and bilateral reducible patellar dislocations. Joint hyperlaxity was also noticed, especially regarding both hips. The spine and pelvis were normal.

Genetical tests were performed when the patient was 11 and confirmed the KAT6B gene mutation which however was missing in both parents. The precision about the mutation was NM_012330.3 (KAT6B): c.4205_4206delCT, classified as OMIM# 603736.

Although his knees were unstable, they allowed a functionally normal femorotibial axis in extension. Conservative management plan was implemented and consisted of physiotherapy, foot-ankle orthosis to correct foot deformity, especially on the left side, and knee orthosis to stabilise limbs axis during standing and walking. He used a K-walker up to age 8. The orthosis was withdrawn at age 12.

**Figure 1:**
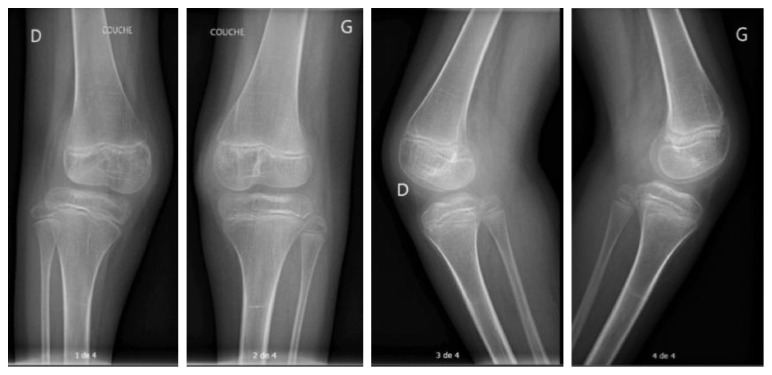
Patient 1: knee X-rays at follow-up (age 15).

After 11 years of observation, the patellar dislocation had evolved from reducible to permanent, inducing a femorotibial subluxation with tibia external rotation in flexion (Figure 1). There was a discrete valgus in extension but enough stability for standing and walking. The left foot axis was normal.

### 2.2. Case 2

The second patient is the same age and was followed since age 3 as well (Table 1). He had a mask-like face with blepharophimosis and ptosis, dental anomalies, and psychomotor delay. His musculoskeletal anomalies were initially bilateral: crossed toes and valgus hindfoot, patellar hypoplasia, femorotibial subluxation, and excessive femoral anteversion. His spine and pelvis were normal.

A genetic workup confirmed the KAT6B gene mutation in the patient, but the parents were not tested. The mutation was classified as OMIM# 603736 with the description that follows: NM_012330.3(KAT6B): c.4775_4794dupCCACGCTCGACGATTGCCA.

Initial management was conservative. It included physiotherapy, verticalization by NF-Walker and parapodium, KAFO-type leg brace, and seating shell.

**Figure 2:**
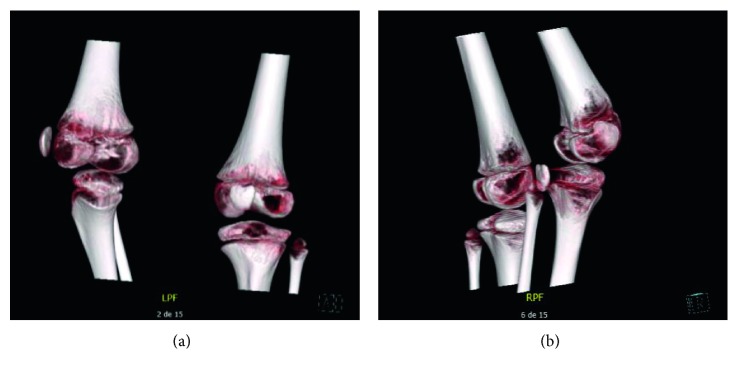
Patient 2: preoperative CT scan 3D reconstructions showing patella subluxation and femorotibial rotation (a) and normal state (b).

At age 7, he could stand up with assistance and make few steps using a NF-walker. At age 11, patellae were palpated in a partially dislocated position on both sides. One year later, left patella remained unchanged but complete and permanent dislocation had occurred on the right side with pain, increased instability, and reluctance to stand on the right lower limb, hence losing the little walking progress he had obtained. Clinical and CT scan (Figure 2) workups of the right knee showed then an extension deficit of about 30°, patella luxation, and tibial external rotation of about 90°.

**Figure 3:**
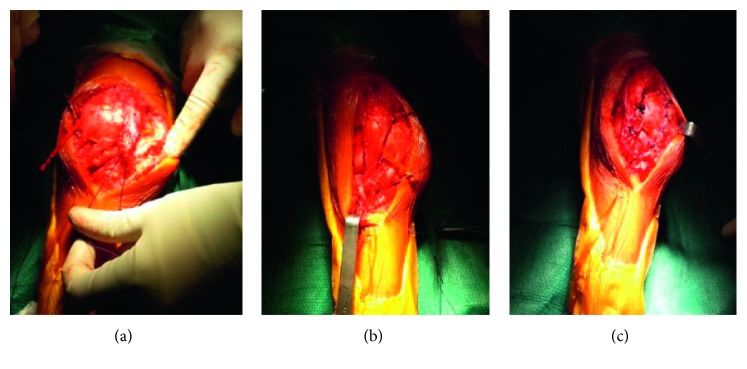
Peroperative pictures: after LLR and MPFL tenoplasty with allograft (a); a portion of vastus medialis was individualised and translocated to the lateral aspect of the patella (Krogius tenoplasty) (b); final sutures (c).

Surgery on the right knee was then proposed in order to regain walking function. It was conducted in two steps: first, an extensive lateral retinaculum release allowing patella reduction, then MPFL double-bundle reconstruction using tendon allograft, associated to Krogius tenoplasty (Figure 3). Peroperative assessment of the knee showed a correctly centered patella and a good range of motion (ROM: 0°–100°).

Postoperative plan was cast for 6 weeks followed by gradual mobilisation.

At cast withdrawal, the ROM quickly deteriorated, resulting in extension deficit of 30° at 8 weeks. Physiotherapy was attempted but remained inefficient at 12 months postoperative.

At this point, botulinum toxin injection was performed in a hamstring muscle group as follows: 450 Units (U) of Dysport® were divided into 3 injections of 100 U in the biceps femoralis and 3 injections of 50 U in semitendinous. We associated cast in maximal extension.

**Figure 4:**
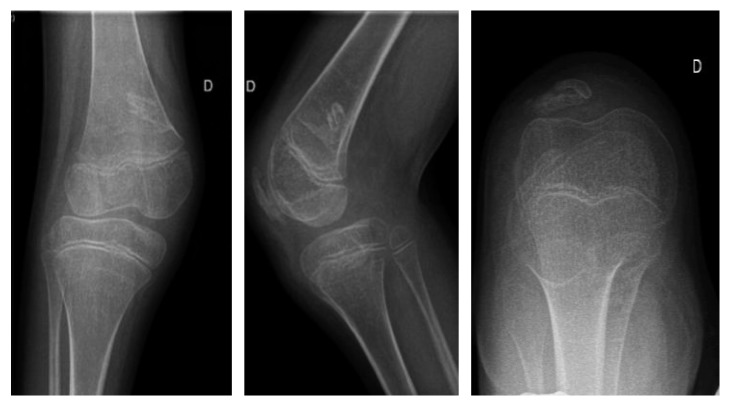
Patient 2: X-ray at follow-up. The patella is centered.

Checkups and cast changing were performed at week 1, week 2, week 4, and week 8 after injection. Extension limitation evolved respectively from −25°, −15°, −8°, to −4°. Standing and walking ability improved along with extension regain. He was able to perform a few steps at week 8. His patella kept a centered position on X-ray (Figure 4) and physical checks at last follow-up.

## 3. Discussion

SBBYS syndrome (OMIM# 603736) is a rare condition. According to Orphanet database's latest report, less than 20 cases have been described in the literature.

The diagnosis is based on clinical features and genetic testing. The clinical workup has been well summarised by Campeau and Lee considering major and minor criteria (Table 2) [3]. Based on this reference, we observed in our both cases at least 2 major criteria and 3 minor criteria, which correlate to the diagnosis of SBBYS variant of Ohdo's syndrome.

Both patients were genetically tested and confirmed a KAT6b gene mutation classified OMIM# 603736, confirming the SBBYS/Ohdo. In one case, parents were also checked and were negative. This is in accordance with recent literature recognising the condition to be caused by de novo dominant mutation of KAT6b gene [4], although rare family recurrence cases exist and are explained by possible gonadal mosaicism [2, 5].

Hypoplastic dislocated patellae were featured in both cases. The management was guided by functional state. The first patient was affected on both sides but with good axial alignment and walking function, which advocated nonsurgical option. The results were good at follow-up.

Conservative treatment with brace and physiotherapy was unsuccessful on the right side in Patient 2 resulting in loss of walking and standing function, which indicated surgical treatment. It was a combined procedure including lateral retinacular release (LRR), double-bundle medial patellofemoral ligament (MPFL) reconstruction, and a vastus medialis tenoplasty according to the Krogius technique. Trochleoplasty and tibial tubercle transposition were not considered because physis was still open.

Isolated LRR has failed to show long-term benefit in case of patellar instability. It has then been suggested as an adjunct to patellar realignment procedures [6].

MPFL is the most important medial structure to control patellar stability throughout flexion as it has been shown to contribute more than 50% of forces that prevent lateral displacement of the knee extensor mechanism. Medial soft structure reconstruction is then crucial in patellar realignment surgery [6].

In our case, MPFL reconstruction was impossible without LRR. Despite the possibility of the single-bundle technique to minimize the number of tunnels in the hypoplastic patella, we decided to use the double-bundle MPFL technique because it has proven better efficiency [7].

Krogius tenoplasty as sole procedure has been reported to be ineffective in cases with joint laxity [8]. In our case, it was useful in filling the lateral defect created by LRR.

The combination of serial casting and toxinum botulinum injection has shown good results in children with cerebral palsy [9]. We referred to this principle to manage the extension deficit of our Patient 2, and it resulted in good improvement after 8 weeks.

## 4. Conclusion

We have observed that in a case of SBBYS variant of Ohdo's syndrome, patella dislocation can be treated either conservatively or surgically with satisfying results. Considering functional preservation, bilateral dislocation can keep good function with conservative treatment, but unilateral dislocation can necessitate surgical correction to maintain or acquire walking and standing abilities.

## Figures and Tables

**Table 1:** Patients' data.

Case	Birth	Age at 1st consultation (year)	Skeletal features	Management	Age at operation	Postop follow-up	Global follow-up (year)	Results
1	2002	3	Bilateral patella dislocation	Nonsurgical	—	—	11	Satisfactory walking function
2	2002	3	Unilateral patella dislocation	Nonsurgical and surgical	13	14 months	11	Satisfactory standing function; starting walking

**Table 2:** Clinical diagnosis guide by Campeau et al. [3].

Category	Features	Likelihood to have the syndrome
Major features	Long thumbs/great toes	(i) Two major features, or
	Immobile mask-like face	(ii) One major feature and two minor features
	Blepharophimosis/ptosis	
	Lacrimal duct anomalies	
	Patellar hypoplasia/agenesis	

Minor features	Congenital heart defect	(i) Two major features, or
	Dental anomalies	(ii) One major feature and two minor features
	Hearing loss	
	Thyroid anomalies
	Cleft palate	
	Genital anomalies	
	Hypotonia	
	Global developmental delay/intellectual disability	
